# Rotational Mechanism Model of the Bacterial V_1_ Motor Based on Structural and Computational Analyses

**DOI:** 10.3389/fphys.2019.00046

**Published:** 2019-02-05

**Authors:** Abhishek Singharoy, Chris Chipot, Toru Ekimoto, Kano Suzuki, Mitsunori Ikeguchi, Ichiro Yamato, Takeshi Murata

**Affiliations:** ^1^School of Molecular Sciences, Arizona State University, Tempe, AZ, United States; ^2^Laboratoire International Associé Centre, Université de Lorraine, Nancy, France; ^3^Department of Physics, University of Illinois at Urbana-Champaign, Urbana, IL, United States; ^4^Graduate School of Medical Life Science, Yokohama City University, Yokohama, Japan; ^5^Graduate School of Science and Molecular Chirality Research Center, Chiba University, Chiba, Japan; ^6^RIKEN Medical Sciences Innovation Hub Program, Yokohama, Japan; ^7^Department of Biological Science and Technology, Tokyo University of Science, Tokyo, Japan; ^8^Precursory Research for Embryonic Science and Technology, Japan Science and Technology Agency, Chiba, Japan

**Keywords:** rotary motor, V-ATPase, X-ray structure, molecular dynamics, free energy

## Abstract

V_1_-ATPase exemplifies the ubiquitous rotary motor, in which a central shaft DF complex rotates inside a hexagonally arranged catalytic A_3_B_3_ complex, powered by the energy from ATP hydrolysis. We have recently reported a number of crystal structures of the *Enterococcus hirae* A_3_B_3_DF (V_1_) complex corresponding to its nucleotide-bound intermediate states, namely the forms waiting for ATP hydrolysis (denoted as catalytic dwell), ATP binding (ATP-binding dwell), and ADP release (ADP-release dwell) along the rotatory catalytic cycle of ATPase. Furthermore, we have performed microsecond-scale molecular dynamics simulations and free-energy calculations to investigate the conformational transitions between these intermediate states and to probe the long-time dynamics of the molecular motor. In this article, the molecular structure and dynamics of the V_1_-ATPase are reviewed to bring forth a unified model of the motor’s remarkable rotational mechanism.

## Introduction

**FIGURE 1:**
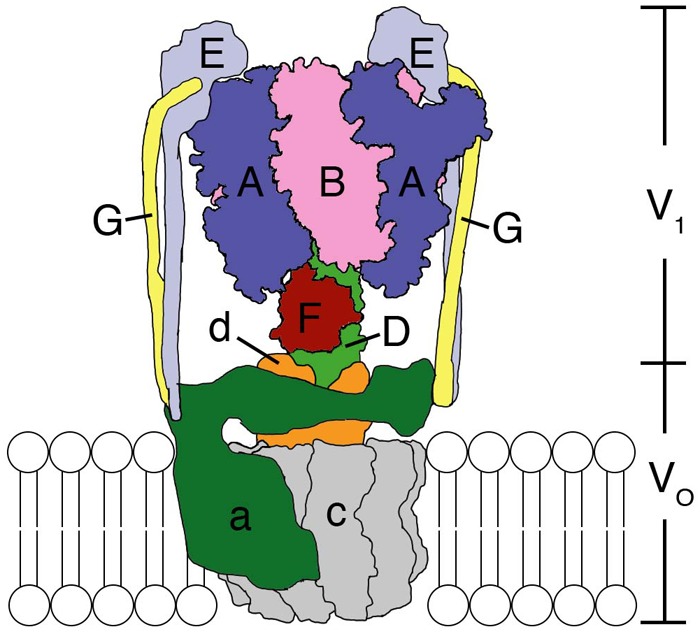
Structural model of the *Enterococcus hirae* V-ATPase. A_3_B_3_ hexamer ring with a DF shaft constitute the V_1_-ATPase catalytic part. The a subunit and c decamer rotor ring constitute the V_o_ membrane-embedded part, which pumps out Na^+^ from the cytoplasm. The EG peripheral stalks and d subunit connect and attach V_1_ and V_o_. For details see text.

The F-, A-, and V-ATPases are unique biological rotary motors, which perform active ion transport by utilizing the energy from ATP hydrolysis (Forgac, 2007). F-ATPase as an ATP synthase functions in the mitochondria, chloroplasts, and oxidative bacteria (Walker, 2013). In archaea, A-ATPase functions as the ATP synthase similar to F-ATP synthase; its structure and subunit composition resemble those of the V-ATPase (Grüber et al., 2014). V-ATPase functions as a proton-transporting ATPase in various organelles, plasma membranes of eukaryotic cells, and bacteria (Kakinuma et al., 1999; Imamura et al., 2005; Forgac, 2007). They consist of a hydrophilic globular catalytic domain (F_1_, A_1_, or V_1_) and a hydrophobic membrane-embedded domain (F_o_, A_o_, or V_o_), which facilitates ion translocation across membranes (Cross and Müller, 2004), as shown in the schematic model of *Enterococcus hirae* V-ATPase (Figure 1).

*E. hirae* V-ATPase functions as a primary ion (Na^+^) pump (Murata et al., 1996, 1999, 2000, 2001, 2005a, 2008) to maintain the homeostasis of intra-cellular ionic environment at high salt concentrations outside providing high salt tolerance to this organism (Kakinuma et al., 1999). It is composed of nine subunits with amino-acid sequences that are homologous to those of the corresponding subunits of eukaryotic V-ATPases (Murata et al., 2001, 2005b; Yamato et al., 2016). V_1_ is composed of an A_3_B_3_ hexameric ring functioning as an ATP hydrolyzing rotary motor, with its DF shaft (Saijo et al., 2011; Minagawa et al., 2013) located inside the A_3_B_3_ ring. A and B are the catalytic and non-catalytic subunits, respectively, which form one catalytic nucleotide-binding A_1_B_1_ pair and the hexameric ring is composed of three such pairs. Single molecule observations revealed that the DF shaft rotates in three 120° steps in a 360° rotation without apparent sub-steps (Minagawa et al., 2013; Ueno et al., 2014; Iino et al., 2015) and the observed dwells are thought to correspond to the catalytic dwell position (Arai et al., 2013). Its rotation speed (∼100 rps at 100 μM ATP) is comparable to that of bacterial F-ATP synthase (Iino et al., 2015). The DF shaft is connected to the c-rotor ring in the membrane via the d subunit (Saijo et al., 2011). The c decamer rotor ring which binds Na^+^ and the a subunit form the V_o_ domain (Murata et al., 2003, 2005a). Na^+^ is believed to be translocated across the membrane through the interface between the a subunit and the c-rotor ring, by utilizing the rotation energy of the c-rotor transmitted via the DF shaft through d subunit (Mizutani et al., 2011). Since a subunit is thought to have two half channels that open to either side of the membrane, a Na^+^ needs to rotate with the c-rotor ring in order to migrate from one half channel to the other (Murata et al., 2005a, 2008; Mizutani et al., 2011). Two peripheral EG stalks are believed to connect the V_1_ and V_o_ domains (Yamamoto et al., 2008). Structural, single-molecule, and computational analyses of the V_1_ and A_3_B_3_ complexes have been extensively conducted to elucidate the rotation catalysis mechanism of the V_1_ rotary motor (Arai et al., 2013; Minagawa et al., 2013; Ueno et al., 2014; Suzuki et al., 2016; Isaka et al., 2017; Singharoy et al., 2017). Here, we summarize such studies and discuss the updated rotation mechanism.

## Crystal Structures of the *E. hirae* V_1_ Motor (EhV_1_)

**FIGURE 2:**
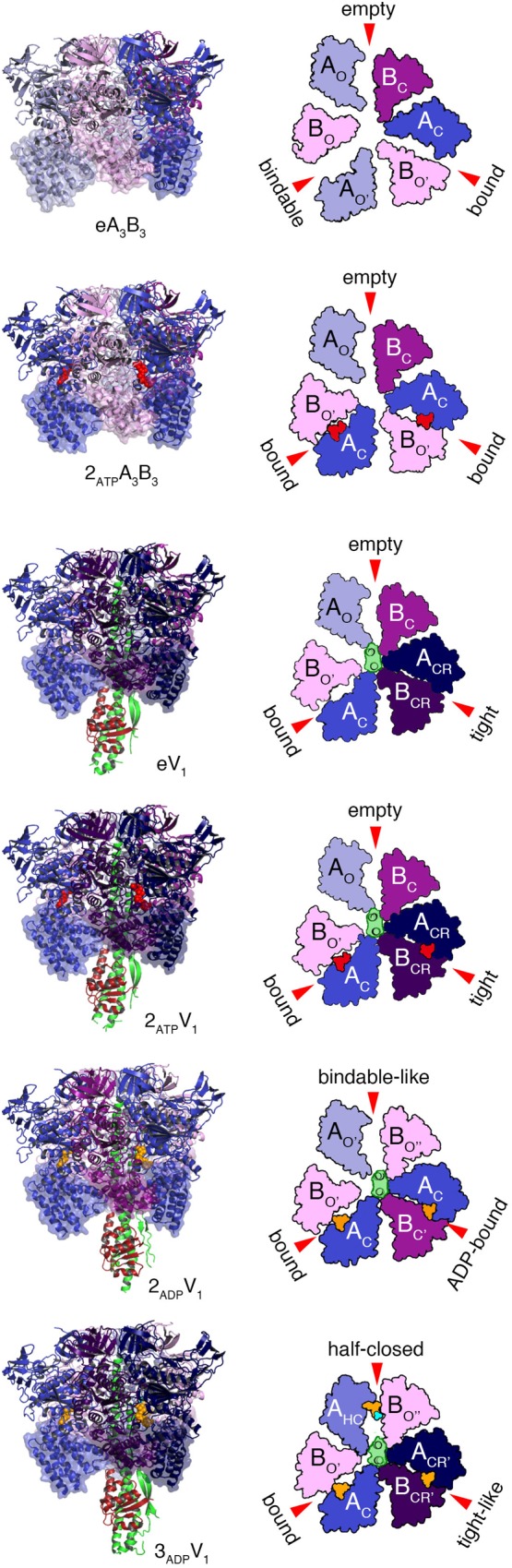
Crystal structures of the A_3_B_3_ and A_3_B_3_DF (V_1_) complexes with or without the nucleotides. Left panels from top to bottom; side views of nucleotide-free A_3_B_3_ (eA_3_B_3_), 2AMP-PNP bound A_3_B_3_ (2_ATP_A_3_B_3_), nucleotide-free V_1_ (eV_1_), 2AMP-PNP bound V_1_ (2_ATP_V_1_), 2ADP bound V_1_ (2_ADP_V_1_), and 3ADP bound V_1_ (3_ADP_V_1_) are shown in ribbon representation. Right panels: top views as observed from the cytoplasmic side of the C-terminal domain (transparent surface in the structures drawn on the left panels) are shown. Open (O and O’; light), closed (C; dark) and closer (CR; darker) conformations of A and B subunits are indicated. D and F subunits of the shaft are shown in green and dark red, respectively. Red arrows indicate the nucleotide-binding sites in the respective forms. The bound nucleotides and sulfate ion are shown in space-filling representation, colored in red (AMP-PNP), orange (ADP), and cyan (SO_4_^2-^).

Various crystal structures of the V_1_ and A_3_B_3_ complexes of *E. hirae* V_1_-ATPase (Figure 2) have been obtained (Arai et al., 2013; Suzuki et al., 2016). The structure of the A_3_B_3_ complex consists of three domains, namely the N-terminal β-barrel domain, tightly connecting the hexamer, the central domain, and the C-terminal domain, forming three nucleotide binding sites. A nucleotide binding site is formed between the A and B subunits functioning as a pair. To examine the structural differences, the C-terminal domains viewed from the N-terminal side are shown in the surface representations, with and without the nucleotides (Figure 2).

The nucleotide-free A_3_B_3_ (eA_3_B_3_) shows a unique asymmetrical structure (Arai et al., 2013); three of the A_1_B_1_ pairs are all in different conformations, with different nucleotide binding affinities, i.e., the ‘empty’ (ATP-unbound form incapable of nucleotide binding; see also the section of ‘free-energy calculations of EhV_1_’), the ‘bindable’ (ATP-accessible form), and the ‘bound’ (ATP-bound form) forms. The asymmetrical structure of eA_3_B_3_ is altered upon binding of the non-hydrolysable ATP analog, adenosine 5’-(β, γ-imino)triphosphate (AMP-PNP). Binding of AMP-PNP to the ‘bound’ and ‘bindable’ forms triggers a conformational change of the ‘bindable’ form to become the ‘bound’ form (2_ATP_A_3_B_3_). These structures suggest that the A_3_B_3_ complex changes its conformation from one asymmetrical structure to another, through the binding and dissociation of the nucleotides in one direction, which determines the order of ATP hydrolysis and in turn should correspond to the rotational direction of the DF shaft.

The structures of eA_3_B_3_ and 2_ATP_A_3_B_3_ change in response to the binding of the DF shaft inside the ring, which results in the formation of a more tightly packed ‘tight’ form, compared to the ‘bound’ form in the A_3_B_3_ complex, with or without the bound AMP-PNP (2_ATP_V_1_ and eV_1_, respectively). The DF shaft binding induced the ‘tight’ form, therefore, the ‘tight’ form is thought to be the major binding form of the DF shaft (Arai et al., 2013). The ‘tight’ form is presumably waiting for ATP hydrolysis to occur during the ‘catalytic dwell’ in the rotary cycles. In this form, the R350 of arginine-finger in the B subunit, believed to catalyze the hydrolysis of ATP, approaches closer to the γ-phosphate of ATP. The other two AB pairs of V_1_ adopt either an ‘empty’ or ‘bound’ form; no ‘bindable’ form is observed, indicating that V_1_ can only bind two AMP-PNP, but not three.

For the hydrolysis reaction to continue, a number of structural changes in the ‘tight’ form need to be induced via the conversion of ATP into ADP and Pi. Crystal structures of the 2ADP-bound (2_ADP_V_1_) and the 3ADP-bound (3_ADP_V_1_) V_1_ complexes have recently been obtained in the presence of 20 μM and 2 mM ADP, respectively (Suzuki et al., 2016). In 2_ADP_V_1_, the ‘tight’ form changes to the ‘ADP-bound’ form, which cooperatively induces a conformational change from the ‘empty’ to the ‘bindable-like’ form; the ‘bindable-like’ form can bind a nucleotide, while the ‘empty’ form cannot. Since electron density of Pi is not observed, even in the presence of 200 μM Pi in the crystallization solution, Pi must have been released soon after ATP hydrolysis at ‘tight’ form, which changes the conformation of 2_ATP_V_1_ to that of 2_ADP_V_1_; 2_ADP_V_1_ is believed to be in the ‘ATP-binding dwell’ state, waiting for ATP to bind. This early release of Pi, in good contrast to the late release in F-ATP synthase as reported (Rees et al., 2012), may be related to their functional differences; F-ATP synthase works as both ATP synthase and ATPase but V-ATPase works specifically as ATP hydrolyzing enzyme. The DF shaft in the 2_ADP_V_1_ does not rotate significantly, but is tilted toward the ‘ADP-bound’ form owing to the conformational changes induced by the binding of ADP to the ‘tight’ form of eV_1_. Such a tilt of the DF shaft without apparent rotation would be difficult to be recognized as an additional sub-step based on single-molecule observations.

The structural differences between the 2ADP-bound (2_ADP_V_1_) and 3ADP-bound (3_ADP_V_1_) V_1_ complexes, which are considered to be induced by ADP binding to the ‘bindable-like’ form of 2_ADP_V_1_, were analyzed. The ‘bindable-like’ form changes to an ‘half-closed’ form. A strong electron-density peak for SO_4_^2-^ (a Pi analog) is observed at the nucleotide binding site with ADP:Mg^2+^ in the 3_ADP_V_1_ complex. The DF shaft and the ‘ADP-bound’ form are slightly attracted to the ‘half-closed’ form; thus, the shifted ‘ADP-bound’ form is rather similar to the ‘tight’ conformation. The nucleotide-binding site is also more similar to that of the ‘tight’ form than that of the ‘ADP-bound’ form. This shifted ‘ADP-bound’ form of 3_ADP_V_1_ is coined the ‘tight-like’ form. The distances between the β-phosphate of ADP and the interacting residues in the ‘tight-like’ form are slightly longer than those in the ‘ADP-bound’ form, suggesting that the binding affinity for ADP of the ‘tight-like’ form must be lower than that of the ‘ADP-bound’ form. Consequently, an ADP molecule will be easily released from the binding site. The structure of 3_ADP_V_1_ is, therefore, believed to correspond to the state of waiting for ADP release (‘ADP-release dwell’) in the rotation. However, since the 3_ADP_V_1_ structure was obtained at an unusually high concentration of ADP (2 mM) for an *E. hirae* cell, the ‘ADP-release dwell’ state might be a minor intermediate state, which might exist in the catalytic cycle with high ADP and low ATP concentrations (Suzuki et al., 2016; Ueno et al., 2018).

## A Rotational Mechanism Model Based on the Crystal Structures

**FIGURE 3:**
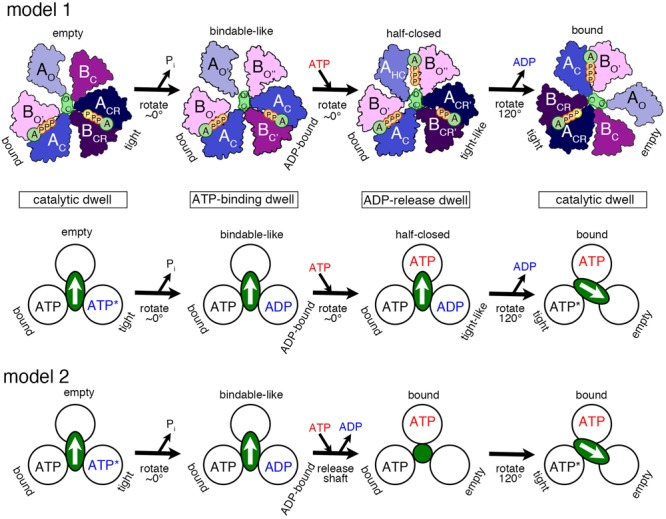
Proposed models of the rotation mechanism of *Enterococcus hirae* V_1_-ATPase based on the crystal structures. [Model 1 (Suzuki et al., 2016)] Upper drawings show the structural models from the left to the right panel based on the crystal structures of 2_ATP_V_1_ (catalytic dwell), 2_ADP_V_1_ (ATP-binding dwell), 3_ADP_V_1_ (ADP-release dwell), and 2_ATP_V_1_. ATP indicated with a yellow ‘P’ in 2_ATP_V_1_ represents an ATP molecule that is committed to hydrolysis. Lower drawing shows the coupling model of the 120° rotation of the shaft (green ellipse with white arrow) and the ATP hydrolysis based on the structural model (upper drawings). Each circle represents the conformation of the nucleotide-binding site, viewed from the cytoplasmic side. The orientation of the shaft begins from the 12 o’clock position in the catalytic dwell waiting for ATP hydrolysis. ATP^∗^ represents an ATP molecule that is committed to hydrolysis. ATP^∗^ is hydrolyzed to produce ADP and Pi, and the Pi release induces the conformational changes to the ATP-binding dwell state, without a rotational sub-step. ATP binding at the ‘bindable-like’ form in the ATP-binding dwell state, induces the conformational changes to the ADP-release dwell, without an apparent rotational sub-step. ADP release from the ‘tight-like’ form induces the dissociation of the shaft, thermal 120° rotation, and consequent conformational changes to the catalytic dwell. [Model 2] An alternative coupling scheme of the model 1 without the ADP-release dwell state is shown. ATP binding to the ATP-binding dwell state induces the concomitant release of the shaft and ADP. Therefore, this transient intermediate structure may correspond to that of 2_ATP_A_3_B_3_ with the shaft (green circle) thermally fluctuating. For details, see text.

Based on the various structures of A_3_B_3_ and V_1_ obtained with or without the nucleotides, we propose a chronology of the main events occurring during one ATP hydrolysis and 120° rotation (Figure 3, model 1 and model 2) as follows:

1. ‘Catalytic dwell’ state: ATP bound to the ‘tight’ form is ready to be hydrolyzed, which produces the products, ADP and Pi.
2. ‘ATP-binding dwell’: The product, Pi, is released first. Then the ‘ADP-bound’ form with the bound ADP is produced from the ‘tight’ form, which in turn induces the conformational change of the ‘empty’ form to the ‘bindable-like’ form. The ‘empty’ form cannot bind a nucleotide; however, due to this conformational change, the ‘bindable-like’ form is accessible for the next ATP. This structure is, therefore, referred to as the ‘ATP-binding dwell,’ waiting for ATP binding. In *E. hirae* V_1_, there is no sub-step observed, but the DF shaft alone appears to tilt slightly.
3. ‘ADP-release dwell’: ATP is bound to the ‘bindable-like’ form, which may first drive the rotation of the shaft, or cause the release of ADP from the ‘ADP-bound’ form. The two events can be either sequential or concomitant; this is not yet revealed by the structural studies.

In regard to the question of how the shaft rotates, several hypotheses have been put forth, such as the typical push–pull mechanism (Kinosita et al., 2004) and a type of thermal ratchet mechanism (Yamato et al., 2016, 2017). In *E. hirae* V_1_, the DF shaft rotates 120° in one step and the traveling distance of the amino acids of the shaft to interact with the motor ring subunits during such 120° rotation in one step appears too long to dissociate from the previous motor subunits and reach the next ones through a push–pull mechanism. A thermal ratchet mechanism, therefore, appears to be utilized by V_1_ instead of a push–pull one.

### Proposed Model 1

Binding of ATP to the ‘bindable-like’ form may induce the conformational transition to the ‘half-closed’ form, which then induces the change of the ‘ADP-bound’ form to the ‘tight-like’ form observed in the 3_ADP_V_1_ structure as the intermediate state during the catalytic cycle. This structure seems to possess a lower affinity for ADP in its ‘tight-like’ form, thus, facilitating the release of ADP. ADP release probably induces the conformational change of the ‘tight-like’ form to the ‘empty’ form, sequentially inducing further conformational change of the ‘half-closed’ form to the ‘bound’ form. During the conformational change, after ADP release, the DF shaft probably dissociates and rotates 120° thermally to the next position.

### Proposed Model 2

Instead, we think that rotation of the shaft starts prior to ADP release. The binding of ATP to the ‘bindable-like’ form in the ‘ATP-binding dwell’ structure may initiate the release of the DF shaft and the conformational change to the ‘bound’ form, which concomitantly induces the release of ADP from the ‘ADP-bound’ form, to produce the ‘empty’ form. In this scenario, the ‘ADP-release dwell’ structure plays no role and instead, A_3_B_3_ with the bound nucleotides (2_ATP_A_3_B_3_) is the probable intermediate structure for this transient step (Arai et al., 2013). The ‘ADP-release dwell’ could be a by-product structure of the ADP inhibited state, or an intermediate during the reverse reaction of ATP synthesis. It has not been yet proven whether the structure shown in the figure under the ‘ADP-release dwell’ is the intermediate structure for this step of ATP hydrolysis.

4. The shaft rotates to the next ‘bound’ form to induce the conformational change to the ‘tight’ form, resuming the original ‘catalytic dwell’ state waiting for ATP hydrolysis.

As discussed above, the aforementioned third step has two optional possibilities (sequential or concomitant), which are not confirmed yet. Furthermore, the structure of 3_ADP_V_1_, as postulated in the above third step, can be a real intermediate of ATP hydrolysis/rotation or an artificial ADP inhibition product observed in the presence of high ADP concentration; this should also be clarified. Computational approaches are expected to be powerful and fruitful to resolve these unsettled aspects.

## Multiscale Molecular Dynamics Simulation of EhV_1_

**FIGURE 4:**
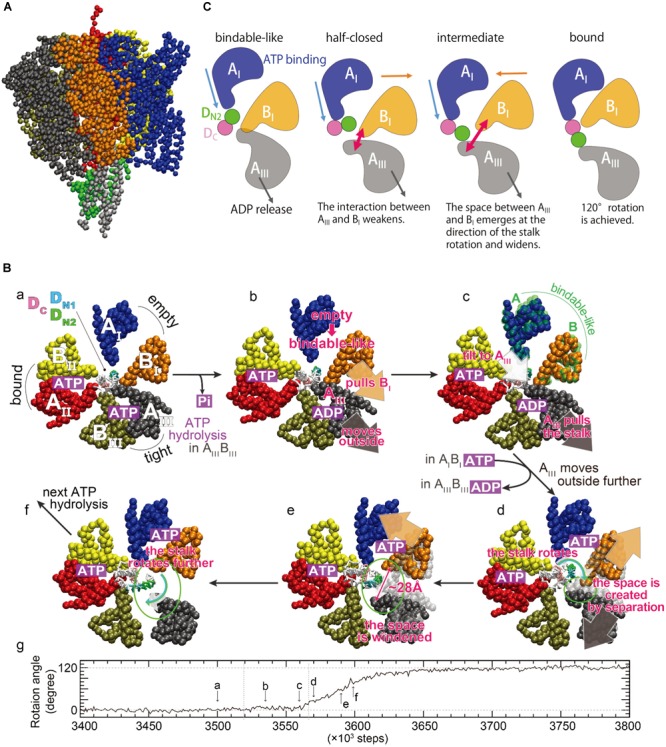
Rotation mechanism proposed by multiscale molecular dynamics simulations. **(A)** A coarse-grained model of V_1_-ATPase. The color coding of each subunit in the A_3_B_3_ ring is the same as that of **(B)**, sub-panel a. The central shaft is colored by gray (D subunit) and green (F subunit). **(B)** Time evolution of the rotation angle of the shaft and typical conformations in a simulation. The sub-letters a–f represent each snapshot, pointing in the direction of rotation angle progression (Isaka et al., 2017). The three AB subunits are denoted as A_I_B_I_ (blue and orange), A_II_B_II_ (red and yellow), and A_III_B_III_ (black and tan), and are the same as A_O_B_C_, A_C_B_O′_, and A_CR_B_CR_ (Figure 2) at the initial structure described in sub-panel a. The region of the shaft enclosed in the A_3_B_3_ ring consists of two helices of the N- and C-terminus of the D subunit, denoted as D_N_ and D_C_ (magenta), respectively. D_N_ is further decomposed into D_N1_ (T20 – L29, cyan) and D_N2_ (K30 – Q39, green). **(C)** A schematic picture of dynamical rearrangements of the AB subunits and the shaft (Ekimoto and Ikeguchi, 2018). The A_I_, B_I_, A_III_, D_N2_, and D_C_ are the same as those in **(B)**. The unidirectional arrows show the movements of the subunits, and the bidirectional arrows show width of the space between A_III_ and B_I_ subunits schematically.

To directly investigate the large-scale and long-time dynamics, such as the DF shaft rotation coupled to the motions of the A_3_B_3_ ring in a straightforward fashion, multiscale molecular dynamics (MD) simulations were conducted using an approach combining a coarse-grained (CG) model with all-atom MD simulations (Isaka et al., 2017). A CG model of V_1_-ATPase was built from the catalytic-dwell crystal structure [2_ATP_V_1_, PDB ID: 3VR6 (Arai et al., 2013)], and one amino-acid residue was represented by one bead located on its Cα atom (Figure 4A). Nucleotides and solvent molecules were not explicitly included in the CG model. We employed the Gō potential [the atomic interaction based CG model (Li et al., 2011)], in which the nucleotide-binding states were implicitly taken into account through the subunit structures. To optimize the CG parameters, the fluctuation of CG residues around a minimum of the Gō potential used in CG-MD simulations was matched to those of all-atom MD simulations at equilibrium near the 2_ATP_V_1_ crystal structure, using the fluctuation-matching methodology (Isaka et al., 2017). Tuning of the CG parameter in terms of protein fluctuation is important to simulate conformational changes induced by ligand binding, because, according to the picture rendered by linear-response theory (Ikeguchi et al., 2005), structural changes upon ligand binding occur as a response to the equilibrium fluctuation of the ligand-free state. Using the tuned CG parameter, the shaft rotation was examined using a multiple-Gō model (Okazaki et al., 2006; Okazaki and Takada, 2008; Yao et al., 2010; Kenzaki et al., 2011), in which two minima were set at the structures, one before and one after ATP hydrolysis, corresponding to the 2_ATP_V_1_ crystal structure and its 120°-rotated structure, respectively.

CG-MD simulations revealed structurally essential features underlying the DF shaft rotation at the CG residue resolution. Several CG-MD simulations have produced a successful 120° shaft rotation with no sub-step (panel g in Figure 4B), consistent with that observed in single-molecule experiments (Iino et al., 2015). A 120° rotation in ∼100 × 10^3^ steps may approximately correspond to a sub-millisecond regime, because a 120° rotation is completed within 0.2 ms according to single-molecule experiments (Iino et al., 2015). Typical conformations during shaft rotation are illustrated in panels a–f of Figure 4B, alongside the time-evolution of the rotation angle. Here, the three AB subunits are termed A_I_B_I_, A_II_B_II_, and A_III_B_III_ (panel a in Figure 4B), and they adopt the ‘empty,’ ‘bound,’ and ‘tight’ structures before the rotation, respectively. From the intermediate structures and conformational changes during rotation, two key structural features were identified: The first is that the A_I_B_I_ pair spontaneously adopts the ‘bindable-like’ structure observed in the 2_ADP_V_1_ crystal structure just before the beginning of the shaft rotation (panel c in Figure 4B). Because the ‘bindable-like’ structure was not used as the input structures, this spontaneous emergence is not trivial. The formation of the ‘bindable-like’ structure was observed in all examined simulations; however, the emergence of the ‘bindable-like’ structure is not the only requirement for successful shaft rotation. In several simulations, shaft rotation did not occur spontaneously due to steric hindrance between the shaft and the B_I_ subunit. Said differently, the B_I_ subunit acts as a gate, and the shaft can pass through the gate by the creation of space between the A_III_ and the B_I_ subunits, i.e., the ‘open-gate’ conformation is achieved. The second structural feature is that the separation of the A_III_ and the B_I_ subunits from each other avoids the steric hindrance during rotation (panels d and e in Figure 4B). The maximal width of the gate was ∼28 Å, and such large openings are not observed in the crystal structures.

A possible mechanism underlying the 120° shaft rotation was proposed on the basis of the CG-MD simulations (Figure 4B). Although nucleotides were not included in the CG model, their binding states could be estimated from the conformational changes of the three AB subunits. ATP hydrolysis and Pi release occurs in the A_III_B_III_ pair, and the A_III_ subunit moves outward (panels a and b in Figure 4B). The A_III_ subunit interacts with both the B_I_ subunit and the shaft, and then the A_III_ subunit pulls them outward, inducing the separation of the A_I_B_I_ pair and a tilt of the shaft (panel b in Figure 4B). Owing to the outside movement of the B_I_ subunit, the A_I_B_I_ pair undergoes a conformational change to the ‘bindable-like’ structure (panel c in Figure 4B). The emergence of the ‘bindable-like’ structure is reasonable because the incoming ATP can bind to the ‘bindable-like’ structure, which has a more open interface than the ‘empty’ structure as described in the previous section. When ATP binds to the ‘bindable-like’ structure, a closing motion of the A_I_B_I_ pair from the ‘bindable-like’ structure to the ‘bound’ structure is induced. In addition, when ADP release occurs at the A_III_B_III_ pair, the A_III_ subunit moves further outward. Consequently, both the outward movement of the A_III_ subunit and the closing motion of the A_I_B_I_ pair cause a separation of the A_III_ and the B_I_ subunits from each other (panels c–e in Figure 4B). Owing to their movements in opposite directions, the separation becomes large, resulting in the creation of a space, i.e., opening the gate enough to avoid steric hindrance between the shaft and the B_I_ subunit. Finally, the shaft passes through the gate (panels e and f in Figure 4B), and then the gate closes, coupled with the closing motion of the A_I_B_I_ pair. The closed gate prevents reverse rotation. In this mechanism, the cooperatively rearranging motions among the AB subunits play a crucial role.

Analysis of interaction patterns among the AB subunits and the shaft provide insight into the mechanism by which the dynamical rearrangements of the AB subunits propagate the shaft (Figure 4C). The region of the shaft enclosed in the A_3_B_3_ ring consists of two helices of the N- and C-terminus, denoted D_N_ and D_C_, respectively. At the initial state, only the A_I_ subunit is in contact with D_N_. After the emergence of the ‘bindable-like’ structure, the shaft is tilted, coupled with the outward movement of the A_III_ subunit. The tilt of the shaft triggers the rotation, and causes an inward shift of the interface between the A_I_ subunit and the shaft. Coupling with the closing motion of the A_I_ subunit itself, the A_I_ subunit approaches the D_C_ and then comes into contact with both the D_N_ and D_C_, just before the open-gate conformation. Such a contact pattern is similar to that between the half-closed structure and the shaft observed in the 3_ADP_V_1_ crystal structure (Suzuki et al., 2016). After the open-gate conformation, the shaft enters into the space between the A_III_ and the B_I_ subunits and rotates, coupling to the further outward motion of the A_III_ subunit and the closing motion of the A_I_ subunit. At the final state, the A_I_ subunit is in contact with the D_C_ only. In summary, three events cooperatively contribute to the shaft rotation: (i) the closing motion of the A_I_ subunit pushes the shaft, (ii) the outward motion of the A_III_ subunit pulls the shaft, and (iii) the open-gate conformation allows the shaft to rotate.

An important difference between this mechanism and the rotation model inferred from a series of crystal structures (model 1 in Figure 3) is the A_III_B_III_ pair, which is the adjacent pair of the ‘half-closed’ structure. As described above, the A_III_ subunit should move outward in order to avoid the steric hindrance. Therefore, the A_III_B_III_ pair should adopt the open (‘empty’) structure, implying that the conformations of the three AB pairs in the 3_ADP_V_1_ crystal structure do not emerge together during a successful 120° rotation.

## Free-Energy Calculations of EhV_1_

**FIGURE 5:**
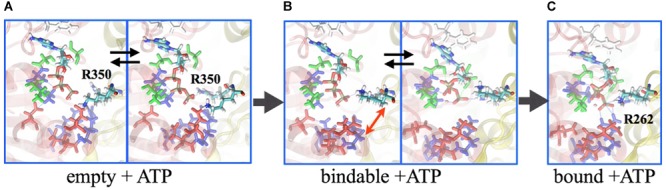
Binding mechanism of ATP. Molecular dynamics simulation reveal that the ATP is excluded from binding to the ‘empty’ site, as the arginine-finger 350 residue sterically prevents the nucleotide entry **(A)**. In the ‘bindable’ site, isomerization of R350 allows entry of ATP **(B)**, ensuing interaction with R262 to ensure binding **(C)**. Labeled in green are residues Q503 and N504, which together with F425 (labeled in white) interact with the adenosine ring; E261 and E262 are labeled in red; G235, G237, and K238 are indicated in blue. Subunit A is presented in transparent red ribbon, and B in yellow.

Central to the investigation of how the V_1_-motor operates is the underlying free-energy change that characterizes, on the one hand, the energy source, i.e., ATP, and, on the other hand, the conformational transition, i.e., the motor action. Based on the studies of Boyer on the so-called binding-change model for the rotational catalysis in F-type ATP synthase (Hutton and Boyer, 1979), which was demonstrated by Gao et al. (2003), employing molecular simulations of the F_1_ domain, a similar approach was taken for V_1_. In particular, the binding affinities of the nucleotides (ATP or ADP.Pi) have been determined employing the alchemical free-energy perturbation (FEP) methodology between the ‘tight,’ ‘bound,’ and ‘empty’ pockets at the AB interface. The binding affinities of ATP to the ‘tight,’ ‘bound,’ and ‘empty’ sites are 11.6 kcal/mol, 8.9 kcal/mol, and 4.1 kcal/mol, respectively, and that for ADP.Pi is 8.9 kcal/mol at the ‘tight’ site and 4.3 kcal/mol at the ‘empty’ site (Singharoy et al., 2017). Thus, the chemical energy in terms of these binding-affinity differences to be utilized by the AB protein subunits to undergo conformational transitions and the central DF shaft to rotate is estimated to be 11.6 kcal/mol (ATP affinity to the ‘tight’ state) – 4.3 kcal/mol (ADP.Pi affinity to the ‘empty’ state) = 7.3 kcal/mol. Importantly, in the ‘empty’ site, there is minimal interaction between the R262 residue of the A subunit and the terminal phosphate of the ATP, since the conformation of the R350 residue on the B subunit prevents entry of ATP into the pocket (Figure 5). Consequently, ATP-affinity to the ‘empty’ site is the least.

The chemical energy produced due to ATP hydrolysis in an aqueous solution is readily dissipated in the environment. However, the same event occurring at protein–protein interfaces induces binding-affinity changes due to side-chain reorganization of the binding pockets, a process that occurs at a much slower timescale. Thus, the binding-affinity changes resulting from ATP hydrolysis potentially serve as a design principle that ATPase employs to prevent dissipation and to channel the chemical energy into mechanical work. Complementing the aforementioned FEP calculations, the binding affinity changes derived from single-molecule experiments, as a function of shaft rotation, reveal that a binding pocket undergoes a cycle of the tight → empty → bound transition during the 120° rotation of the shaft, during which the pocket experiences energy changes of 5–6 kcal/mol over the millisecond timescale (Adachi et al., 2012), much slower than the picosecond scale of energy dissipation of ATP hydrolysis in an aqueous solution.

Conversion of the chemical work into mechanical work is captured by employing a second type of free-energy methodology, namely geometric transformation schemes (Chipot and Pohorille, 2007). The mechanical changes within a chemically charged V_1_ following the hydrolysis step is probed using string simulations with swarms of trajectories (Pan et al., 2008). Combination of the FEP and the string methodologies offers a general theoretical framework for capturing a nanoscale motor in action (Singharoy and Chipot, 2017; Benson et al., 2018). Energy changes along the most probable conformational transition pathway in V_1_, underlying the rotation of the central shaft as a mechanical response to ATP hydrolysis, product (ADP.Pi) release, and binding of a new reactant ATP was found to be approximately 6 kcal/mol (Singharoy et al., 2017).

## Conformational Rotation Pathway of EhV_1_

Qualitative examination of the simulations performed employing the string method with swarms of trajectories of the entire V_1_ (Singharoy and Chipot, 2017; Singharoy et al., 2017) did not reveal any significant difference in the conformation of the motor structures determined by crystallography (Arai et al., 2013). Analysis of the sequence of events characterizing the conformational transition in V_1_, however, unveiled additional, subtle, albeit key milestones, absent in the structural studies.

**FIGURE 6:**
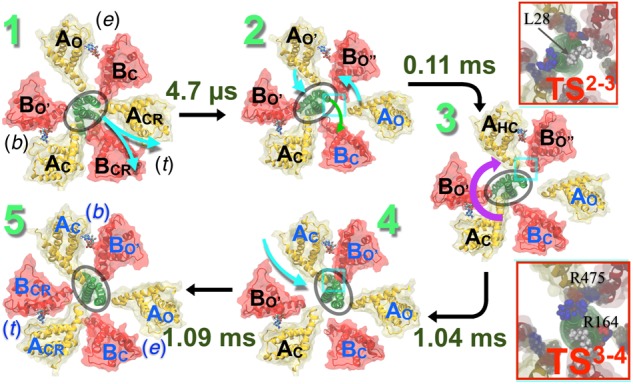
Conformational transitions mechanism of V_1_ proposed by computer simulation studies. Pathway of the hydrolysis-driven conformational transition in the entire V_1_ domain derived from the string method with swarms of trajectories (Pan et al., 2008). Firstly, the transition from a ‘tight’ (t) to an ‘empty’ (e) conformation (1→2) is observed at the ADP+Pi-bound site, where A_CR_B_CR_ is transformed to A_O_B_C_. This transition promotes ATP binding at the neighboring ‘empty’-site. This empty site transforms from the A_O_B_C_ conformation in state 1 to a bindable A_O′_B_O″_ form in state 2. ATP-binding to the bindable site yields the first doubly bound state (2→3); the ATP binding simultaneously induces a local deformation of the DF-shaft. The shaft then rotates yielding the second doubly bound state (3→4). Finally, a bound ATP (b) in the third site, in A_C_B_O′_ form, transitions to the reaction mode in the ‘tight’ (t) conformation, promoting subsequent hydrolysis (4→5). Rates of each of these transitions are computed employing techniques described in Singharoy and Chipot (2017) and Singharoy et al. (2017) and labeled along with each step. The rotation step is found to be the slowest when it follows product release. (Upper inset) The 2→3 transition necessitates a wring deformation of the shaft that marginally exposes the hydrophobic residue L28 to water. This unfavorable solvation characterizes the first TS (TS^23^). (Lower inset) Salt bridges between residues of the shaft and those of the A_HC_ and B_O″_ subunits reorganize during the shaft-rotation step, involving transient repulsive electrostatic interactions of residues R164 (DF) with R475 (A_HC_). These repulsive interactions characterize the second TS (TS^34^), featuring the highest barrier of the V_1_–rotation pathway. Blue beads indicate basic residues, red indicates acidic and white indicates hydrophobic ones. Cumulative transition times are recorded at each step: transition 1→2 takes 4.7 μs, 1→3 takes 0.11 ms, 1→4 takes 1.04 ms, and altogether transitions 1→5 takes 1.09 ms. Figure adapted from Singharoy et al. (2017).

Similar to the crystallographic structures (Figure 6), first, the ‘tight’ conformation transforms into the ‘empty’ form, which prefaces further opening of the adjacent ‘empty’ interface, transforming the latter into a ‘bindable’ site. The newly formed ‘bindable’ site provides more open conformations than the ‘empty’ site, facilitating access of a loosely bound ATP to the ATP-binding residues (binding affinity 4.3 kcal/mol). The stated tight → empty transformation also weakens the AB-DF interface, allowing the bent shaft to straighten. Second, upon ATP association to the ‘bindable’ site of the A subunit, the corresponding interface closes through a hinge-bending motion to a ‘bound’ state. This bindable → bound transformation induces a wringing deformation on the straightened central shaft at the locus, where it interacts with the newly formed ATP-bound A subunit. Third, the wrung shaft rotates by 120°, featuring two ‘bound’ and one ‘empty’ state. Lastly, the ‘bound’ site already occupied by ATP evolves into the ‘tight’ form completing the rotatory catalysis mechanism in V-type ATPase. The bound → tight transformation induces a bend on the 120°-rotated shaft, reestablishing its adhesive contact with the ‘tight’ interface.

A second notable observation in agreement with the crystallographic data is that, at any point across the pathway, the nucleotide (ATP or ADP.Pi) binding ability of the binding pocket in the A subunit is correlated with the A-shaft interaction: the ‘empty,’ the ‘bound’ and the ‘tight’ sites with the lowest, intermediate and highest ATP-binding affinity, respectively, belong to domains that characterize minimal, primarily electrostatic, and combined electrostatic and hydrophobic interactions with the shaft.

Finally, a critical comparison of the simulations incorporating and devoid of the central shaft confirms that in the absence of the latter, the A_3_B_3_ ring is looser and more prone to energy dissipation, albeit still capable of catalytic activity, thus reinforcing the idea that the DF domain improves dramatically energy-conversion efficiency. Such loose AB interfaces have also been observed in the crystal structures of the isolated A_3_B_3_ bereft of the shaft, furnishing a third point of agreement between the computational and crystallographic findings.

## Similarities and Differences Between the Pathways

A key, yet justifiable discrepancy between the experimental and the computational models of rotational catalysis in V-type ATPases stems from the dynamical property of the central shaft. Given that the shaft is always devoid of deformation in the various crystal structures, which represent the local free-energy minima of the conformational landscape of ATPase motor-action, it is quite intuitive to assume that the shaft rotates as a rigid-body (Arai et al., 2013). Refining this idea, the simulations reveal that the central shaft within V_1_ first undergoes a wringing transformation, followed by the rotation of the deformed shaft, and finally restores its configuration in the rotated state. Indeed, at either end of the rotation, the simulations predicted, in line with the structural models, that the shaft remains bereft of deformation. However, the pathway revealed deformability of the shaft—a finding that is consistent with single-molecule experiments, which evince the possibility of energy storage within a shaft due to its inherent elasticity (Sielaff et al., 2008; Martin et al., 2018).

Deformability of the central shaft inculcates a key design principle by virtue of which the overall time of the rotation step occurs over a millisecond timescale. Because the shaft rotates in layers and not as a whole, a larger barrier of rotation is split into smaller barriers, which can be overcome in a more biologically relevant timescale. A cumulative transition time of 1.09 ms is estimated for the ATP-binding and 120°rotation (Figure 6). This time should be corrected by the diffusive ATP hydrolysis-product, i.e., ADP.Pi, release time, which was estimated in an independent study to be ∼2.6 ms (Okazaki and Hummer, 2013). Thus, one complete 360° rotation is expected to take 3 × (2.6 + 1.09) = 11.07 ms. These rate estimates add up a rotation speed of about 90.3 s^-1^, which is in good agreement with the single-molecule measurement of an average rotation rate of 89–115 s^-1^ (Ueno et al., 2014).

## Combined Rotational Mechanism Model

**FIGURE 7:**
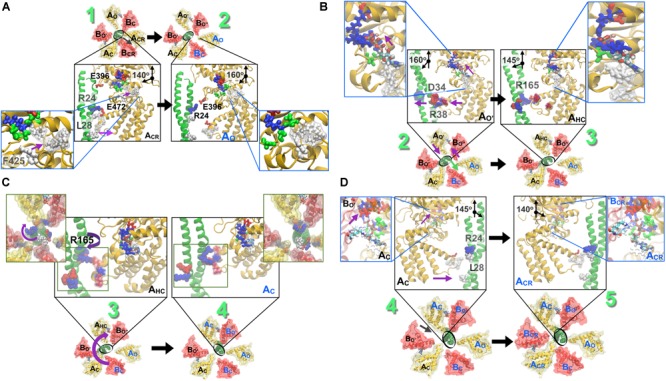
Stepwise breakdown of the rotation mechanism captured in string simulations. **(A) 1→2 transition**. Outward swivel of the A subunit, by 20°, during the A_CR_ → A_O_ transition is allosterically coupled to the motion of F425 in the ATP binding pocket. L28 (also presented in Figure 6) becomes solvent exposed. **(B) 2→3 transition.** Inward swivel of the A subunit by 15° during the A_O′_ → A_HC_ transition is allosterically coupled to the motion of F425 in the ATP binding pocket (inset). As ATP crawls into the ‘bindable’ site (Figure 5), F425 forms a π–π stacking interaction with the adenine base, which is accompanied by an inward swiveling motion of the A_O′_. **(C) 3→4 transition.** Rotation of the DF-shaft follows to reinstate the lost interactions of several residues. First, the R194 from the shaft binds to the E347 from A_O_ following which E180 and R14 from the shaft combines with R271 of B_O′_ and E352 of A_C_ and thereafter residues E161, R164 and R165 from the shaft reorganize to be stabilized by R475 of the A_HC_, E384 of B_O″_ and E472 of A_HC,_ respectively. **(D) 4→5 transition.** Inward swivel of the A subunit, by 15°, during the A_C_ → A_CR_ reinstates the salt-bridge interactions between R24 from the shaft and E472 from A_CR_. As in Figure 6, blue beads indicate basic residues, red indicates acidic, and white indicates hydrophobic ones. Figure adapted from components in Singharoy et al. (2017).

A combined rotational mechanism based on the crystal structures (Figure 3) and the computer simulation studies (Figure 4, 6, 7) is summarized as described below.

### Transition From the Catalytic Dwell to the ATP-Binding Dwell

As shown in Figure 3, the ATP bound to the ‘tight’ form is hydrolyzed, producing ADP and Pi. Pi should be first released. The ‘tight’ form changes to the ‘ADP-bound’ form, which cooperatively alters the conformation of the ‘empty’ form to the ‘bindable-like’ form, to which ATP is accessible. In CG-MD simulations, the ‘bindable-like’ form spontaneously emerged (Figure 4B) despite the fact that the ‘bindable-like’ form was not used as an input structure. Widening of the AB interface from the ‘empty’ form is induced by the outward motion of the B subunit pulled by the adjacent A subunit, which undergoes an outward motion upon Pi release. As illustrated in Figure 7A, starting from the tightly bound state of the AB interface bereft of ATP, a similar induction of the conformational change to a ‘bindable’ form was produced in the all-atom string simulations. Displacement of key residue F425 initially exposed to water results in a hinge-bending motion of the A subunit, which swivels outward, leading to the so-called ‘empty’ state, concomitant with the straightening of the central shaft. This concerted motion is accompanied by the replacement of the salt bridge formed by residues R24 and E472, respectively, of the DF shaft and the A_O_ subunit, by that of residues R24 and E396, and by a reorganization of the hydrophobic packing. Outward swiveling of the A subunit triggers a similar movement of the B subunit, thereby converting the neighboring AB interface into a ‘bindable’ state.

### Transition From the ATP-Binding Dwell to the ADP-Release Dwell

As depicted in Figure 3, ATP is first bound to the ‘bindable-like’ form. Following Model 2 or scenario 2, binding of ATP to the ‘bindable-like’ form initiates the release of the DF shaft, and the conformation of the ‘bindable-like’ form changes to the ‘bound’ form, which induces the release of ADP from the ‘ADP-bound’ form, thereby producing the ‘empty’ form. The resultant structure corresponds to the 2_ATP_A_3_B_3_.

According to the string simulation depicted in Figure 7B, as ATP diffuses into the binding site, residue F425 forms a π-stacking interaction with adenine, concurrent with inward swiveling of the A subunit and the conformation transition of the B subunit, which slides toward A to optimize the contacts with the nucleotide (Figure 7B). In CG-MD simulations, although nucleotides were not included in the simulations, the shift of the interaction pattern between the DF shaft and the A subunit, that might be resulted from the conformational change described above, was observed (Figure 4C) and induced DF shaft rotation.

### DF Shaft Rotation

Crystallographic studies do not supply dynamical information on the shaft rotation. In *E. hirae* V_1_, the DF shaft rotates by 120° in one step; the traveling distance of the amino acids of the shaft to interact with the motor ring subunits during such one step 120° rotation appears too long to dissociate from the previous and reach the next subunits by a push–pull mechanism. We, therefore, believe that a thermal ratchet mechanism is functional.

Complementing the experiments, the string simulation study of the V_1_ motor gives a detailed picture of the functioning of the DF shaft rotation. In brief, wringing of the central shaft ensues in response to the space (Figure 4B, 6) liberated as the A and B subunits slide toward each other, accompanied by a modification of the partners implicated in the salt bridges established across the A and DF shaft (Figure 7C). Reformation of the disrupted salt bridges as a consequence of the wrung central shaft is only partial, and requires further rotation of the latter to restore the lost interactions. It is noteworthy that reorganization of the interactions at play in the course of the 120° catalytic cycle occurs in a concerted fashion with the deformation and the rotation of the DF shaft, exploiting its elastic characteristics. Swiveling of the A subunit, for instance, triggers the deformation of the upper part of the central shaft, followed by the rotation of its lower part. In CG-MD simulations, the exchange of the interactions described above was observed as an exchange of contacts among the A subunits and the D_N_ and D_C_ parts of the DF shaft (Figure 4C).

### Transition From the ADP-Release Dwell to the Catalytic Dwell

Finally, as shown in Figure 3, the DF shaft rotates to the next ‘bound’ form to induce a conformational change to a ‘tight’ form, resuming the original ‘catalytic dwell’ state waiting for ATP hydrolysis. The simulations describe in more detail the dynamical movement of this transition (Figure 7D). In this last step toward the final state of the V_1_–motor, ATP diffuses deeper within the binding site, allowing it to interact with residue E261, which is the key to initiate hydrolysis (Figure 7D). Further swiveling of the A subunit brings the latter to a tightly bound state, germane to reinstate the disrupted salt bridge between residues R24 and E472, and the DF shaft to bend toward the AB interface, thereby completing the allosteric transition of the A_3_B_3_ ring.

## Conclusion and Perspectives

Static snapshots and dynamical simulation pictures of the V_1_ rotary motor from *E. hirae* are presented and compared in this review article. Simulation studies provide a complementary view of the rotation and ATP hydrolysis, by connecting the static intermediate structures during rotation. Most of them are consistent and complementary: After the ‘empty’ form changes to the ‘bindable’ form, new ATP is bound to induce further conformational changes to drive the shaft rotation, which appears to undergo a wringing movement during rotation. However, certain parts are inconsistent, perhaps due to the insufficient structural information, or suboptimal simulation strategy. The unique asymmetry of the A_3_B_3_ ring with three identical A_1_B_1_ pairs is in line with its meta-stable structure. The mechanism of forming such asymmetrical meta-stable structure will be elucidated in the near future. Further biochemical, crystallographic, and long-time atomic-scale simulation studies will clarify the basic principles of the chemo-mechanical coupling mechanism of the rotary motor, transitioning between such meta-stable structures.

## Author Contributions

All authors discussed findings, analyzed literature, and wrote the manuscript.

## Conflict of Interest Statement

The authors declare that the research was conducted in the absence of any commercial or financial relationships that could be construed as a potential conflict of interest.
